# Mapping scholarly continuing professional development publications in the *South African Family Practice* journal (2020–2025): Trends, gaps, and a call for contributions

**DOI:** 10.4102/safp.v68i2.6293

**Published:** 2026-03-31

**Authors:** Mareike Rabe, Ramprakash Kaswa, Klaus B. von Pressentin

**Affiliations:** 1 Vita Oncology, Cape Town, South Africa; 2 Department of Family Medicine and Rural Health, Walter Sisulu University, Mthatha, South Africa; 3 Division of Family Medicine, Department of Family, Community and Emergency Care, Faculty of Health Sciences, University of Cape Town, Cape Town, South Africa

**Keywords:** continuing professional development, family medicine, primary healthcare, medical education, South Africa

## Abstract

Continuing professional development (CPD) comprises self-directed educational activities that are vital to the lifelong learning of healthcare professionals. It maintains competence, adapts to new guidelines, and supports evidence-based practice. In South Africa, professional boards mandate CPD, which is offered via journals, webinars, courses, conferences, and digital platforms. We evaluated CPD articles in the *South African Family Practice* (SAFP) journal from 2020 to 2025. Clinical content dominated, with guidance on infectious and chronic diseases. However, online engagement was limited. This 6-year snapshot, part of the journal’s 45-year celebration, emphasises SAFP’s role in advancing primary care education. It also highlights opportunities for greater inclusivity and accessibility, for innovation in CPD publishing, and the need for future evaluations of readership patterns and practitioners’ needs.

**Contribution:**

This article reviews CPD publishing in SAFP over 6 years, focusing on themes, authorship, and engagement challenges within its 45-year legacy of promoting lifelong learning in primary care.

## Introduction

### Background: The South African continuing professional development landscape

To meet evolving community health needs, healthcare professionals (HCPs) must engage in self-initiated lifelong learning that sustains and advances their knowledge, skills, values, and practice. Continuing professional development (CPD) encompasses formal and informal activities that build on initial training to maintain competence, prepare for new roles, and enhance professional effectiveness.^1,2,3^

Their respective professional boards mandate South African HCPs to comply with CPD rules and regulations under Section 26 of the *Health Professions Act No. 56 of 1974*.^4^ Healthcare practitioners are required to complete a minimum total of continuing education units (CEUs) over 12 months across the domains of clinical practice, ethics, human rights, and health law, with the total number of CEUs depending on their professional board.^5^ A wide range of platforms and opportunities exist to support CPD for HCPs. Locally, the South African Academy of Family Physicians (SAAFP; ‘the Academy’ for this article) provides accredited CPD through the *South African Family Practice* (SAFP; ‘the journal’ for this article), monthly webinars and online modules and courses hosted on the AOSIS eCPD® platform. Academic institutions and professional organisations also offer structured courses, workshops, and conferences, while a range of digital platforms (e.g. MYCPD,^6^ Focus on Health,^7^ MediXeed,^8^ deNovoMedica^9^) provide accessible online learning opportunities tailored to comply with the Health Professions Council of South Africa (HPCSA). National initiatives include the Department of Health’s Knowledge Hub,^10^ which curates webinars and training resources for HCPs across disciplines. Internationally, organisations such as the World Organisation of Family Doctors (WONCA) and the International Primary Care Associations (IPAs) facilitate global exchange through congresses, collaborative networks, and online resources. In addition, pharmaceutical companies and independent providers contribute CPD through symposia, case-based modules, and specialist updates. These opportunities span both in-person and online formats, enabling HCPs to engage at local, national, and international levels according to their professional needs and contexts.

The SAFP, a peer-reviewed journal that aims to provide primary care providers, researchers, and educators with a broad range of published work on practice, training, and learning, has been involved in CPD activities since its inception in 1980.^2,3^ In 1999, when obligatory CPD was introduced, the HPCSA accredited the Academy as a CPD provider: an acknowledgement of the substantial expertise in general and family practice education that the Academy had built over its first two decades.^2,3^ Key figures include Professor Julia Blitz, who chaired the CPD Task Team, secured the Academy’s CPD accreditation, and established the operational systems that positioned it as a leading accreditor, and professors Pierre de Villers and Gboyega Ogunbanjo, who served as both CPD Editors and Editors-in-Chief of the journal.^3,11,12^

At the time of writing, the journal hosted 537 CPD articles available to readers on the publisher’s online platform.^13^ The journal, which previously published six parts annually, now produces four. On average, it publishes 12 peer-reviewed CPD articles each year, with approximately three addressing non-clinical contemporary healthcare needs, aiming for three articles per part or quarter. Our goal is to ensure diverse representation among academic institutions by balancing author contributions. This involves pairing discipline-specific content specialists with family physicians or primary care experts, taking into account varying levels of professional experience and expertise to promote comprehensive, balanced perspectives throughout the publication process. To earn CPD points, readers must register on the AOSIS eCPD platform, navigate to the eCPD® Healthcare section, review pertinent SAFP articles, and pass a quiz with at least 70%. They have three attempts to succeed and earn three CEUs, supporting continuous professional growth and development. Members of the Academy can register and enrol in courses free of charge as part of their membership. In contrast, non-SAAFP members pay an annual fee to access the platform.

The SAFP editorial team previously published an overview of the original scientific research published during 2020–2022.^14^ During this period, 117 original research manuscripts were published, covering the Southern African quadruple disease burden (non-communicable diseases, including mental health, communicable diseases, maternal, newborn, and child health). At the time of the previous review, there were more than 4200 citations and 768 600 downloads (2018–2023), and the journal’s CiteScore was 1.1. At the time of writing this CPD review, there are over 6300 citations, 2.7 million downloads, and the CiteScore has increased to 2.0 in 2024 (Scopus preview – Scopus – South African Family Practice).

Following the above 2023 review, the editorial team undertook a similar exercise focusing on the CPD articles published in the journal during 2020–2025, to plan and map potential content contributions for future CPD publishing cycles.

## Mapping the CPD content: Our approach

We conducted a cross-sectional descriptive review of CPD content published from 2020 to 2025. A Google spreadsheet was used to capture the relevant data, which was reviewed for accuracy, and the team assisted in interpreting and presenting the descriptive findings.

## *South African Family Practice* CPD content: A 6-year snapshot

During 2020–2025, 74 CPD articles were published. Of these, 56 articles (76%) were categorised as clinical content, while 18 articles (24%) were classified as ethics-related content, addressing issues of professionalism, leadership, and educational spheres of practice.

**FIGURE 1:**
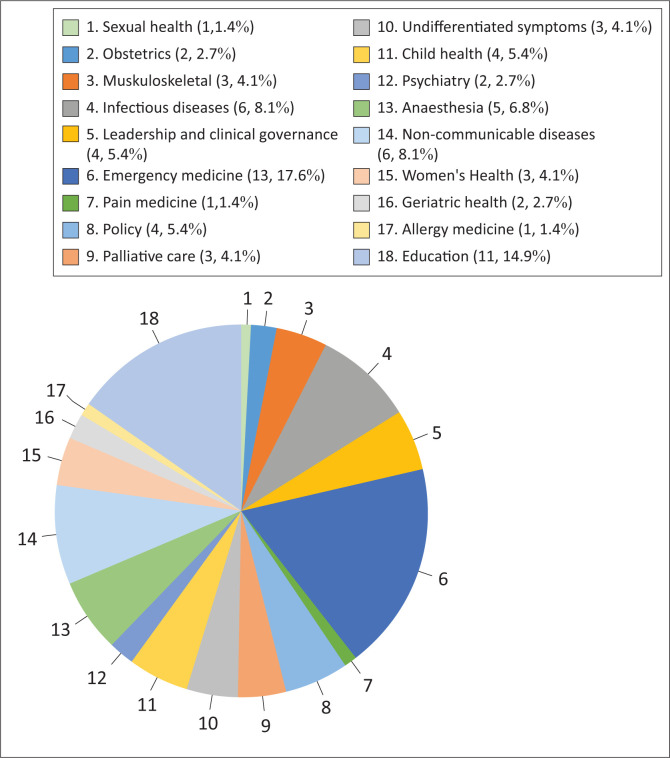
Continuing professional development domains for the period 2020–2025.

Figure 1 depicts the different clinical and non-clinical domains covered. Thirteen articles (17.6%) addressed emergency medicine topics, including a series of three articles on vascular access across the life course for HCPs working in primary emergency care settings. Eleven articles (14.9%) had overarching educational aims, ranging from general practice guidance, such as how to use interpreters effectively in primary care settings, to educator-focused content, such as setting high-quality multiple-choice questions for clinical medicine evaluation. All other domains accounted for less than 10% of the content. Two of the six articles focused on infectious diseases, entitled *Primary care management of the coronavirus (COVID-19)* and *Novel coronavirus pandemic: A clinical overview* were published on 31 March 2020 and 26 June 2020, respectively, reflecting the journal’s commitment to offering timely guidance to readers in the face of novel and emerging diseases. Non-communicable disease content shared the same proportion of publications as infectious diseases (*n* = 6; 8.1%) and covered topics such as insulin use in type 2 diabetes mellitus, Ramadhan fasting for people living with chronic illness, and an approach to heart failure in the public sector, reflecting the journal’s commitment to addressing specific populations commonly encountered in primary care in South Africa. Articles on leadership and clinical governance (e.g. the contribution of primary care HCPs to community-oriented primary care), policy (covering multiple legislative topics in the South African context), and child health (e.g. developmental delay) all constituted 5.4% (*n* = 4) of CPD articles published. The domains of obstetrics, musculoskeletal problems, pain medicine, palliative care, undifferentiated symptoms, psychiatry, women’s health, geriatric health, allergy medicine, and sexual health all constituted less than 5% of published CPD articles.

Table 1 provides an overview of the distribution of CPD authors across academic institutions represented during 2020–2025. A total of 126 authors contributed to the published CPD articles, with an average of 1.7 authors per article (range 1–4). The University of KwaZulu-Natal (UKZN) was the only institution with more than 20% representation (*n* = 26) across all authors and first-author categories, demonstrating its consistent and valuable contribution to this section of the journal. Authors from Walter Sisulu University (WSU) and the University of Cape Town (UCT) contributed equally to the all authors category (*n* = 17; 13.5%). Still, the WSU authors accounted for a larger proportion of first authors (*n* = 10; 13.5%) than those from UCT (*n* = 7; 9.5%). The University of the Witwatersrand (Wits) is also well represented, with 9.5% (*n* = 12) of all authors and 14.9% (*n* = 11) of first authors. Of the 10 academic institutions with medical schools in South Africa, Nelson Mandela University (NMU) and the University of Limpopo (UL) had the fewest authors, with two and one, respectively (neither represented a first author).

**TABLE 1:** Distribution of continuing professional development authors across academic institutions and organisations represented during 2020–2025.

Academic institutions and organisations represented	All authors (*n*)	All authors (%)	First authors (*n*)	First authors (%)
University of KwaZulu-Natal	26	20.6	20	27.0
Walter Sisulu University	17	13.5	10	13.5
University of Cape Town	17	13.5	7	9.5
University of the Witwatersrand	12	9.5	11	14.9
Stellenbosch University	11	8.7	7	9.5
University of Pretoria	9	7.1	2	2.7
Sefako Makgatho University	9	7.1	6	8.1
Private Practice	8	6.3	5	6.8
University of the Free State	6	4.8	3	4.1
Outside South Africa	3	2.4	1	1.4
Nelson Mandela University	2	1.6	0	0.0
Rural Doctors Association of South Africa	1	0.8	1	1.4
North-West University	1	0.8	0	0.0
University of Fort Hare	1	0.8	0	0.0
University of Limpopo	1	0.8	0	0.0
University of South Africa	1	0.8	0	0.0
Durban University of Technology	1	0.8	0	0.0
South African Medical Association	1	0.8	0	0.0

**Total**	**126**	**100.0**	**74**	**100.0**

## Discussion

The CPD section of the journal between 2020 and 2025 was dominated by clinical content, with emergency medicine and educational themes most prominent, alongside timely guidance on infectious diseases and attention to chronic disease management. On average, the journal publishes 12 peer-reviewed CPD articles each year, of which approximately three address non-clinical contemporary healthcare needs. Between 2020 and 2025, this pattern was reflected in the publication of 74 CPD articles, of which 56 (76%) were clinically focused, and 18 (24%) explored ethics-related themes such as professionalism, leadership, and education. Authorship was unevenly distributed, with UKZN, WSU, UCT, and Wits accounting for the majority of contributions, whereas newer or resource-constrained institutions such as NMU and UL were under-represented. The strong representation of WSU and UCT may reflect the circles the editorial team moves in, as these institutions are represented on the team. The editors also support emerging researchers and content experts as coauthors to provide scientific writing support and capacity building. The limited contributions of NMU and the UL may reflect contextual and structural factors. Nelson Mandela University’s medical school, established only in 2021, is still developing its academic footprint, while the UL has historically focused on undergraduate training and community-oriented service delivery. Resource constraints, regional disparities, and weaker editorial linkages may further explain their minimal representation in the journal’s CPD publishing. Authors from private practice, institutions outside South Africa, and organisations beyond the country’s 10 medical schools – including North-West University, the University of South Africa, Durban University of Technology, the Rural Doctors Association of South Africa (RuDASA), and the South African Medical Association (SAMA) – make valuable contributions to the journal’s CPD content. However, RuDASA and SAMA may appear under-represented, as some of their members list affiliations with academic institutions when submitting manuscripts rather than with RuDASA or SAMA.

The editorial team currently plans and tracks CPD content using a shared spreadsheet that is updated on an ongoing basis. In designing this content, we strive to distribute it equitably across the domains outlined above, while also considering suggestions and requests from our readership. We also aim to distribute authorship equitably across academic institutions and departments, the private sector, and RuDASA. However, contributions from these groups may be limited, and the team often works pragmatically with the submissions available.

Anecdotally, feedback from sources indicates that eCPD participation among members and paid subscribers remains low, highlighting ongoing engagement challenges. While readers can access CPD articles directly on the journal platform, engagement through the members-online portal – ironically, the space where CEUs are earned – appears restricted. Our current analysis is limited to content that was invited and published, without assessing user engagement or readership patterns. Future evaluations should therefore explore these dimensions more systematically, for example, through membership surveys or interviews with users of the members-online platform. In addition, primary research on the evolving CPD needs of primary care HCPs would be valuable, particularly regarding alternative opportunities such as journal clubs, free webinars, conferences, and other journals, which may represent competing avenues for continuing education.

### Reflections on the way forward

The journal has long maintained a tradition of offering high-quality CPD content, and the previous and current editorial teams have sought to ensure its relevance in the evolving CPD landscape. The journal’s editorial team is committed to ensuring that its CPD publications reflect an equitable balance across the three core pillars of the journal: advancing science, fostering academic development, and promoting social responsibility.

The editorial team is exploring ways to enhance the accessibility of CPD content for journal readers and to ensure that it remains relevant and fit for purpose. We are working with special-interest groups, such as a subset of the Academy responsible for monthly webinars, to streamline content across both forums to reach a wider audience. Conversations are also underway with working groups in oncology, palliative care, and geriatric health to provide more precise, robust, and helpful guidance to HCPs, as these populations are growing, and it is within the purview of primary care HCPs to manage them. We are also in discussion with under-represented institutions, specifically liaising with senior, experienced clinicians to involve more junior researchers and content writers, thereby providing a platform to equip them with skills in academic writing and clinical content creation. Podcasts are emerging as a valuable medium for CPD content creation and dissemination, and the team is currently reviewing the potential of this platform.

Publication fees are waived for CPD articles, and submissions are accepted by invitation only (see Submission guidelines). Authors and reviewers of CPD content earn CPD points from the HPCSA (see Frequently Asked Questions). The editors welcome any suggestions and invite all potential authors to contact them should they wish to submit an article for review. We encourage potential reviewers to volunteer their services, as this is vital to the success of the CPD programme. Please feel free to contact us with your ideas and suggestions on how our journal should develop.

## Conclusion

As the journal celebrates 45 years of promoting family medicine and primary healthcare, this review of CPD content from 2020 to 2025 highlights its ongoing role in supporting lifelong learning and adapting to the changing needs of primary healthcare practitioners and learners. The snapshot reveals a strong emphasis on clinical content and on contemporary non-clinical healthcare needs, and ongoing efforts to balance authorship across academic institutions and sectors, despite enduring challenges. These findings highlight the journal’s dual role: providing accessible, accredited CPD content while also reflecting broader challenges in equity, inclusivity, and practitioner participation. Looking ahead, the editorial team remains committed to enhancing accessibility, diversifying contributions, and adopting new formats, such as podcasts, to ensure that CPD content continues to evolve in step with the profession. In doing so, the journal builds on its legacy while reaffirming its place as a cornerstone of primary care education and professional development in South Africa.
